# Supplementary material for: Rapid Crystallization and Fluorescence of Poly(ethylene terephthalate) Using Graphene Quantum Dots as Nucleating Agents
Source: Polymers (Basel). 2023 Aug 22;15(17):3506. doi: 10.3390/polym15173506 (PMC10490498; doi:10.3390/polym15173506)
Supplement: Supplementary file 1 [file polymers-15-03506-s001.zip › polymers-2501670-supplementary.pdf]

**Rapid Crystallization and Fluorescence of Poly(ethylene terephthalate) Using Graphene Quantum Dots as Nucleating Agent**

Liwei Zhao [1]*, Yue Yin [2], Wanbao Xiao [1], Hongfeng Li [1], Hao Feng [1], Dezhi Wang[1], Chunyan Qu[1]

1 Institute of Petrochemistry, Heilongjiang Academy of Sciences, Harbin 150040, China; zhaoliwei0130@163.com and zhaoliwei0130@hit.edu.cn (L. Zhao); xiaowanbao@126.com (W. Xiao); lihongfengcn@126.com (H. Li); fhbighouse2008@163.com (H. Feng); jim603@163.com (D. Wang); quchunyan168@163.com (C. Qu)

2 Harbin FRP Institute, Harbin 150036, China; yinyue@stu.hit.edu.cn (Y. Yin)

* Correspondence: zhaoliwei0130@163.com and zhaoliwei0130@hit.edu.cn (L. Zhao)

**Non-isothermal Crystallization Process of PET/GQDs Nanocomposites**

Cooling scans of PET/GQDs nanocomposites with different GQDs content are presented in Figure S1. A small number of GQDs (0.25 wt%) have little effect on the crystallization temperature of nanocomposites. Therefore, PET-GQDs will not be discussed in subsequent experiments.

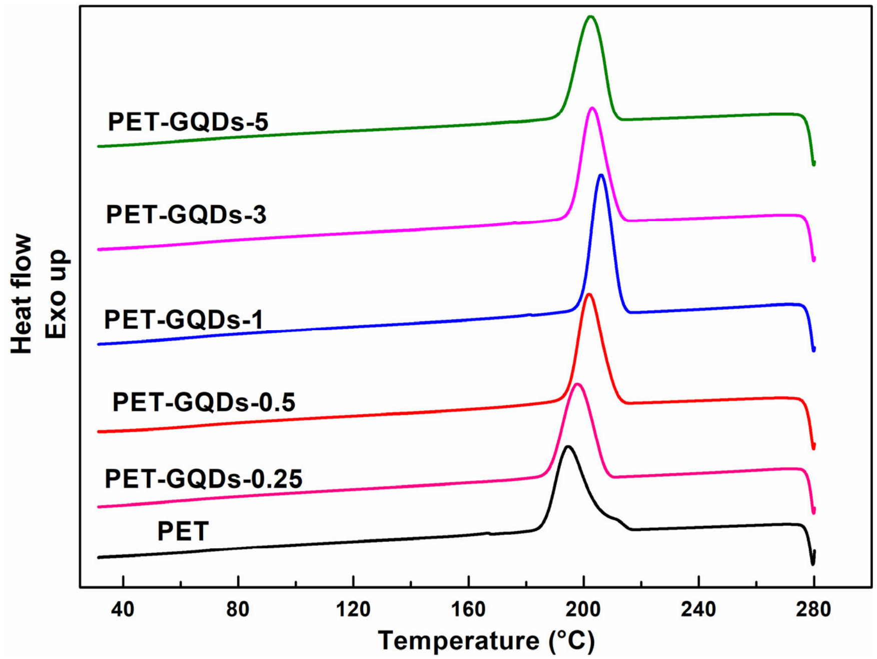


**Figure S1.** DSC curves of PET/GQDs nanocomposites in cooling scans

**Figure S2** shows the non-isothermal crystallization curves of PET and PET/GQDs nanocomposites at a cooling rate ranging from 5 to 20 °C/min. **Figure S2** clearly shows that the addition of GQDs has a significant impact on the crystallization behavior of PET/GQDs nanocomposites. The crystallization peak width of PET/GQDs nanocomposites first narrows and then widens with the increase of GQDs content, while Tc exhibits an initial increase followed by a decrease. Specifically, the crystallization peak of PET-GQDs-0.5 nanocomposites is narrower and higher than that of pure PET, indicating that the addition of 0.5wt% GQDs can result in a shorter and more vigorous crystallization process, which supports the idea that GQDs effectively promote the crystallization rate of PET/GQDs nanocomposites. Interestingly, as the GQDs content increases, the crystallization temperature of PET/GQDs nanocomposites decreases, suggesting that excessive GQDs may hinder the crystallization of PET. Notably, a small

amount of GQDs (0.5 wt%) is sufficient to serve as nucleating agents and promote the crystallization of PET.

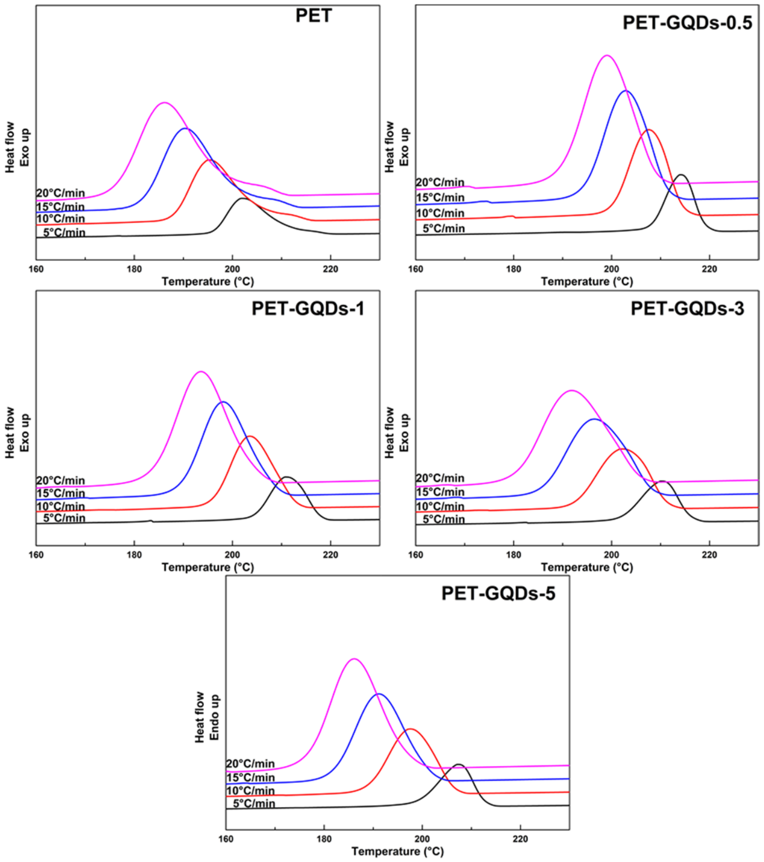


**Figure S2.** Non-isothermal crystallization curves of PET and PET/GQDs nanocomposites at different cooling rates

**Figure S3** depicts the correlation between the relative crystallinity and crystallization time of PET/GQDs nanocomposites. It is evident from the figure that the crystallization time of all samples decreases with an increase in the cooling rate. This phenomenon is attributed to thermal hysteresis, which leads to a longer crystallization time of the nanocomposites with decreasing cooling rates. It is noteworthy that the complete crystallization of PET/GQDs nanocomposites occurs faster than that of pure PET. Furthermore, the different cooling rates have a relatively minor impact on the complete crystallization time of PET/GQDs nanocomposites as compared to pure PET. This implies that GQDs exhibit an excellent heterogeneous nucleation ability in the PET matrix. In addition, the GQDs content in the PET/GQDs nanocomposites has a significant influence on the crystallization behavior of PET. When the GQDs content exceeds 3%, the time required for complete crystallization of PET increases.

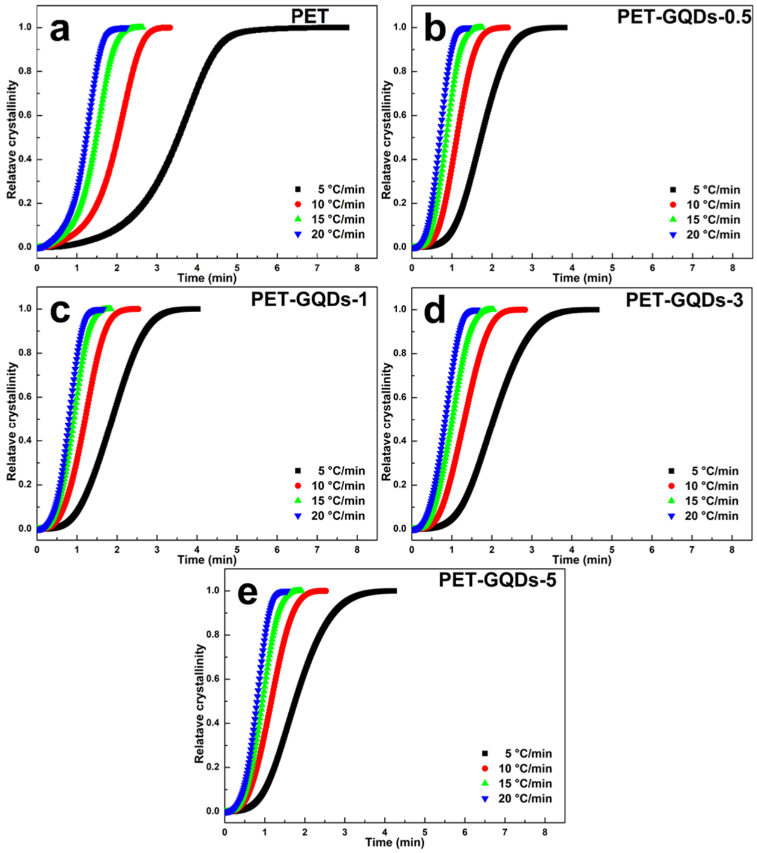


**Figure S3**. Relative crystallinity versus time curve of PET and PET/GQDs nanocomposites at different cooling rate

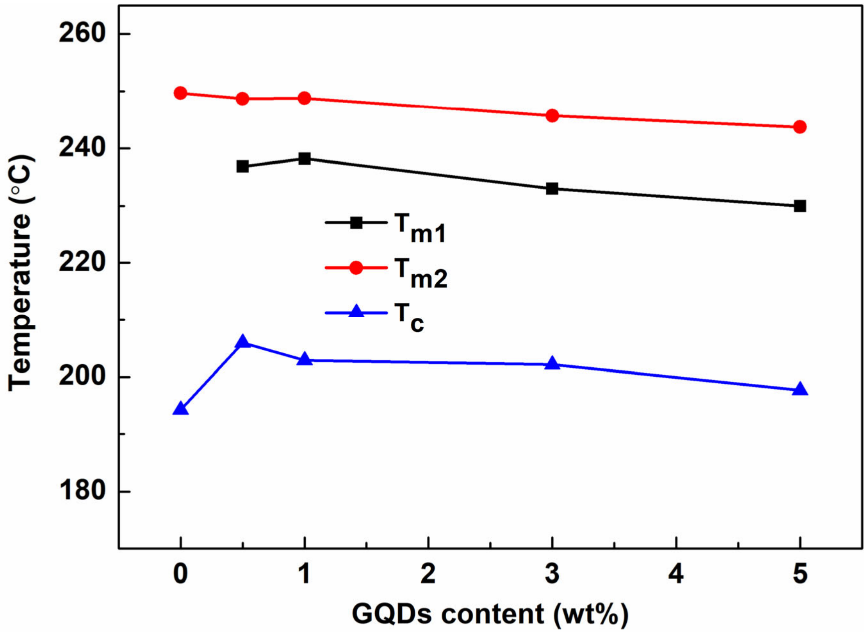


**Figure S4.** Crystallization temperature (Tc) and melting temperature (2nd heating) as a function of the GQDs content

**Non-Isothermal Crystallization Activation Energy of PET/GQDs Nanocomposites**

In **Figure S5**, the temperature ($T$) of PET/GQDs nanocomposites at various relative crystallinity was determined using the Friedman's method. Scatter plots were generated using $\ln(\Phi/T^2)$ and 1/T, and linear fitting was performed on the obtained scatter points. The slope obtained from the fitting represents -ΔE/R, where R is the gas constant. By analyzing the slope, the non-isothermal crystallization activation energy (ΔE) of PET/GQDs nanocomposites at different relative crystallinity levels was determined. These data were further processed and organized in **Figure S6.**

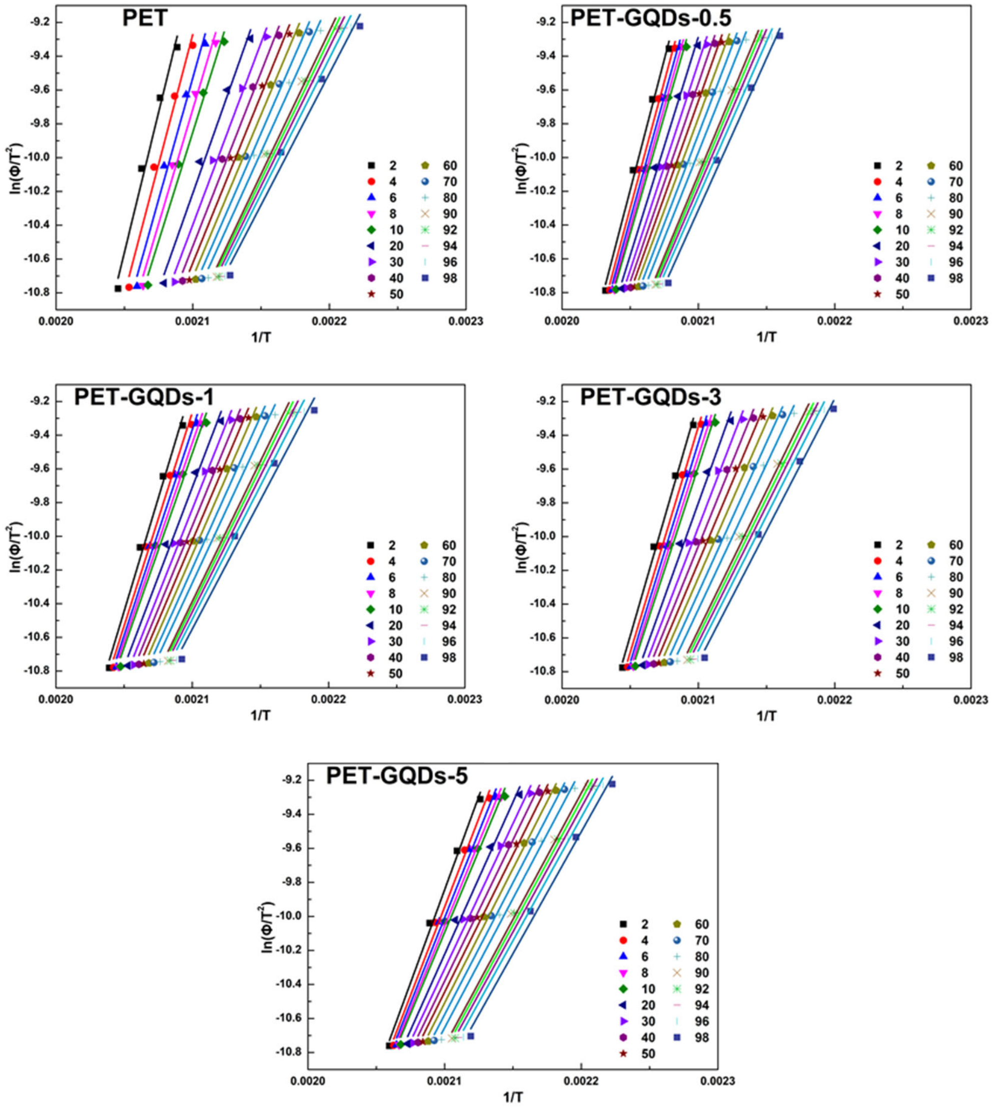


**Figure S5**. The fitting lines of PET/GQDs nanocomposites by Friedman's method

Based on **Figure S6,** it is evident that the activation energy of PET crystallization increases with increasing relative crystallinity, indicating that the crystallization process

becomes progressively challenging. During the initial stage of crystallization, PET crystals can grow freely in the three-dimensional direction, which leads to high crystallization ability and low activation energy. However, as the relative crystallinity increases, the proportion of crystals in the overall material volume increases, thereby inhibiting crystal growth and increasing activation energy.

Furthermore, the addition of GQDs at 0.5wt% significantly reduces the crystallization activation energy of PET/GQDs nanocomposites. This can be attributed to the smaller particle size of GQDs, which provide enough heterogeneous nucleation sites with a lower concentration. However, when the GQDs content increases to 3%, excessive nucleating agents can hinder PET crystallization, resulting in similar crystallization activation energy to pure PET. Although the large number of crystal nuclei can lead to fast crystal growth, when the GQDs content is further increased to 5%, the hindering effect of GQDs on PET crystallization cannot be offset by the number of crystal nuclei, leading to further increasing of the crystallization activation energy.

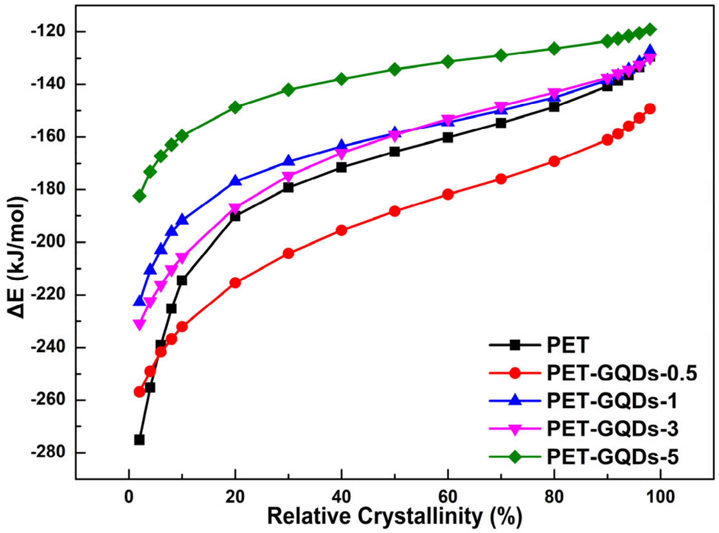


**Figure S6**. The crystallization activation energy of PET/GQDs nanocomposites

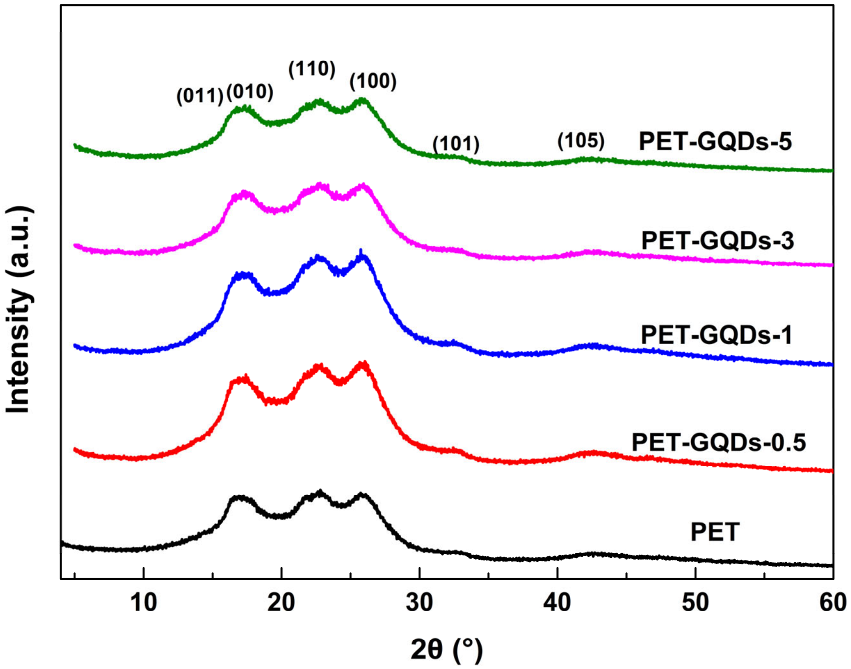


**Figure S7.** XRD patterns of PET and PET/GQDs nanocomposites

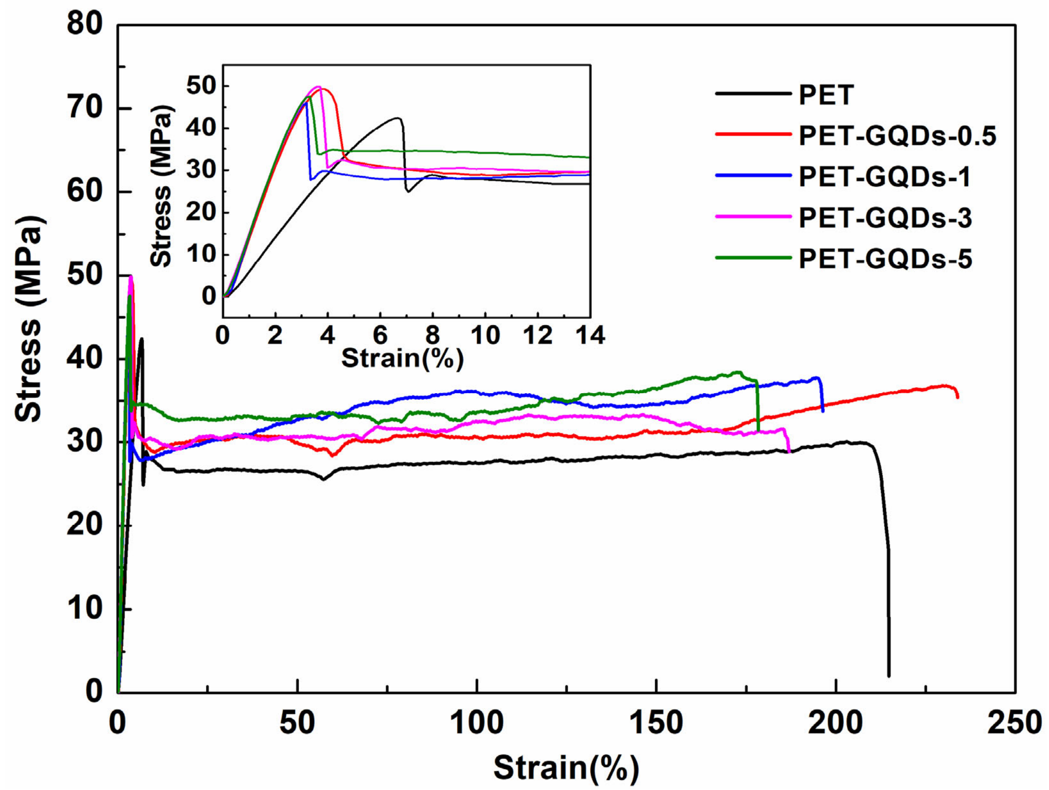


**Figure S8.** Tensile properties of PET/GQDs nanocomposites

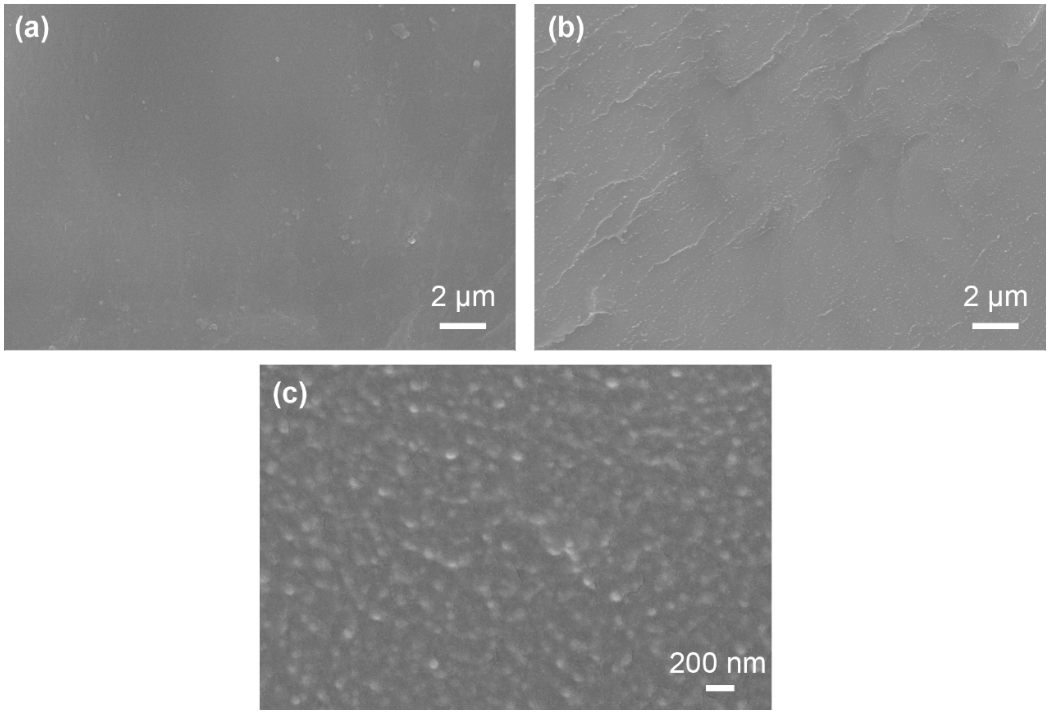


**Figure S9.** SEM images of (a) PET, (b) PET-GQDs-0.5 nanocomposites and (c) high resolution for PET-GQDs-0.5 nanocomposites

Due to the small diameter of GQDs, it is difficult to observe its dispersion effect in the PET matrix through SEM images. Therefore our amorphous samples of PET and PET-GQDs-0.5 were placed in an oven at 125°C for 5 min for crystal growth. As can be seen in Figure S9a, PET is difficult to crystallize at 125°C due to the lack of nucleating agents. Figure S9b and Figure S9c present a homogeneous dispersion of PET-GQDs-0.5 exhibiting a size of nearly 50 nm. The small particles in the image were attributed to the spherulites with GQDs as the nucleus.
